# Duration and Density of Fecal Rotavirus Shedding in Vaccinated Malawian Children With Rotavirus Gastroenteritis

**DOI:** 10.1093/infdis/jiz612

**Published:** 2019-12-13

**Authors:** Aisleen Bennett, Louisa Pollock, Khuzwayo C Jere, Virginia E Pitzer, Benjamin Lopman, Naor Bar-Zeev, Miren Iturriza-Gomara, Nigel A Cunliffe

**Affiliations:** 1 Malawi-Liverpool-Wellcome Trust Clinical Research Programme, College of Medicine, University of Malawi, Blantyre, Malawi; 2 Centre for Global Vaccine Research, Institute of Infection & Global Health, University of Liverpool, Liverpool, United Kingdom; 3 Department of Epidemiology of Microbial Diseases, Yale School of Public Health, Yale University, New Haven, Connecticut, USA; 4 Department of Epidemiology, Rollins School of Public Health, Emory University, Atlanta, Georgia, USA; 5 International Vaccine Access Center, Bloomberg School of Public Health, John Hopkins University, Baltimore, Maryland, USA; 6 National Institute for Health Research (NIHR) Health Protection Research Unit in Gastrointestinal Infections, University of Liverpool, Liverpool, United Kingdom

**Keywords:** rotavirus, transmission, vaccine effectiveness, shedding

## Abstract

Quantifying rotavirus shedding among vaccinated individuals will aid understanding of vaccine indirect effects. Serial stool samples were collected from 196 children who presented with rotavirus gastroenteritis to health facilities in Blantyre, Malawi, and were tested for rotavirus using a VP6 semi-quantitative, real-time polymerase chain reaction. The median duration of fecal shedding was 28 days (95% CI, 19–28). The median copy numbers for peak shedding were 1.99 × 10^7^ (interquartile range, 3.39 × 10^6^ to 6.37 × 10^7^). The fecal viral load was positively associated with disease severity and negatively associated with serum anti-rotavirus immunoglobin A. High and persistent rotavirus shedding among vaccinated children with breakthrough disease may contribute to ongoing transmission in this setting.

## Teaser

Serial stool samples from Malawian children with rotavirus gastroenteritis tested for rotavirus using polymerase chain reaction had high levels of viral shedding despite vaccination. High and persistent rotavirus shedding among vaccinated children with breakthrough disease may contribute to ongoing transmission.

Rotavirus vaccine has been introduced in over 90 countries worldwide, including 45 low-income or GAVI Alliance-eligible countries [1]. However, rotavirus vaccine effectiveness is reduced in low-income countries (LIC) [2], and in several countries rotavirus remains the commonest cause of hospitalized gastroenteritis in children <5 years, despite high vaccine coverage [3]. Vaccine effectiveness is higher against severe rotavirus disease than milder disease [4], but the impact of vaccination on fecal shedding (and therefore infectiousness) amongst vaccinated individuals with breakthrough rotavirus disease is unknown. Differences in the intensity of fecal-oral transmission of rotavirus between settings (force of infection) may contribute to the observed variation in vaccine performance, but few data exist on individual-level fecal shedding, which is the primary mechanism of rotavirus transmission.

The dynamics of fecal shedding of rotavirus in unvaccinated populations has been examined in children with both asymptomatic rotavirus infection and with clinical rotavirus disease using electron microscopy, enzyme-immune assay, and polymerase chain reaction (PCR) [5]. Fecal shedding has been shown to extend beyond symptom resolution, persisting for a median of 10 days after symptom onset in Australia and 24 days in India when using PCR-based assays [6, 7]. A positive relationship between fecal viral load and disease severity was demonstrated among Indian children [5].

There is a lack of shedding data from vaccinated infants in any setting, including infants from sub-Saharan Africa, in whom differences in intestinal integrity, nutritional state, comorbidities, and immune responses may lead to variations in shedding, with concomitant impacts on transmission. We aimed to describe patterns of wild-type rotavirus shedding over time and identify the factors associated with fecal viral load in children age-eligible for vaccination with symptomatic rotavirus disease in Malawi, an LIC in southern Africa. The live oral monovalent rotavirus vaccine was introduced into Malawi’s national immunization program on 29 October 2012, with doses at 6 and 10 weeks of age.

## METHODS

Children age-eligible for vaccination (born on or after 12 September 2012 and >6 weeks old) presenting with acute gastroenteritis to Queen Elizabeth Central Hospital and to 3 government health centers in Blantyre, Malawi, between 16 February 2015 and 11 November 2016 were tested for rotavirus using immunochromatographic tests on a fecal sample. Following informed consent, rotavirus-positive children had demographic data and 2 mls of serum collected at recruitment to measure anti-rotavirus immunoglobin (Ig) A titres; a second bulk stool sample was obtained 48 hours after presentation (primary cohort). A subset of children had more intensive stool sampling carried out for 28 days (intensive cohort); samples were collected daily from the time of presentation for the first 7 days after symptom onset, twice weekly from 7 until 14 days, and weekly from day 14 until day 28.

### Data Collection

Disease severity was defined using the 20-point Vesikari score (<7 indicates mild, 7–10 indicates moderate, and ≥11 indicates severe disease). Severe acute malnutrition was defined by either a weight-for-height Z-score (WHZ) less than -3 standard deviations, a mid–upper arm circumference <115 mm, or nutritional edema [8]. Weights were adjusted for percentage dehydration prior to calculating anthropometric indices. Data on human immunodeficiency virus (HIV) status and vaccine status were collected from government-issued health passports. HIV testing was performed by the government health system. An HIV infection was defined as a positive HIV rapid test (≥12 months of age) or positive HIV DNA PCR (<12 months). Children of HIV-positive mothers were defined as HIV-exposed.

### Laboratory Procedures

Stool samples were tested for wild-type rotavirus using a real-time semi-quantitative reverse transcription PCR [5]. An additional real-time semi-quantitative reverse transcription PCR targeting a distinct rotavirus gene (NSP3) was run on each sample with a cycle threshold value in the range of 35–40 as a confirmatory assay [9]. Due to the lack of reproducibility in samples with very low viral loads, samples were defined as rotavirus positive if they had ≥100 viral copy numbers and were positive on an NSP3 assay.

Serum anti-rotavirus IgA titres were measured using a semi-quantitative sandwich enzyme-linked immunosorbent assay [10]. Results were calculated on a minimum of 2 values per sample with a coefficient of variation <20% and were expressed as geometric mean titres (IU/ml).

### Statistical Analysis

The viral load data did not follow a normal distribution, so were log-transformed for analysis. Risk factors for fecal viral shedding density were investigated using multivariable linear regression, where the outcome variable was the peak log-viral load (the largest value obtained from the 2 samples) in children from the primary cohort. Variables achieving a Wald test *P* value of ≤.1 on a univariate analysis were selected for evaluation in the multivariable model. Nested models were compared using F tests. Variables which improved the model fit (*P* ≤ .05) were retained in the final model.

Data from the intensive cohort were used to evaluate changes in viral load over time using linear mixed models with a random intercept to account for within-child clustering; for this analysis, all data were included, regardless of viral load. Polynomial terms (quadratic and cubic) were included to account for the non-linear relationship between fecal viral load and time.

A time-to-event analysis was used to estimate the duration of viral shedding in children from the intensive cohort. The event of interest was defined as the cessation of shedding and the start time for analysis was the onset of symptoms. Cessation of shedding was defined as the last time point from which rotavirus could be detected until censoring. Thus, an individual with no detectable rotavirus at a given analytical time point but who was shedding rotavirus in subsequent samples was classified as having ongoing shedding at the time of analysis. The follow-up was limited to 28 days.

## RESULTS

We recruited 196 index children, from whom 374 fecal samples were collected (Supplementary Table S1). Of these children, 21 were also recruited into the intensive cohort and had a further 136 samples collected. The median age of all children was 11.5 months (interquartile range, 8.8–15.2). A total of 25 (13%) children were exposed to HIV. Of children with documented HIV test results, 2/58 (3.5%) were infected with HIV. The majority of children had severe rotavirus gastroenteritis (168/193, 86.5%). The median number of anti-rotavirus IgA titres at presentation was 4 IU/ml (range, 0–831), and 27% (45/164) children met the traditional threshold for seroconversion (>20 IU/ml) [10]. The 2-dose rotavirus vaccine coverage was 194/196 (99.0%).

### Predictors of Viral Load: Primary Cohort

The median copy numbers for peak shedding were 1.99 × 10^7^ (interquartile range, 3.39 × 10^6^ to 6.37 × 10^7^). The 2 unvaccinated children had peak viral loads comparable to the vaccinated children (median peak shedding, 9.64 × 10^6^). In a multivariable analysis, a positive association between peak shedding density and Vesikari score and a negative association between peak shedding density and IgA titres were identified (Table 1). The reference group used for Vesikari (mild disease) contains only 3 observations; however, the relationship between Vesikari score and viral load persisted when Vesikari was coded as a continuous variable or as 3 approximately equal groups (Supplementary Table S2). There was also weak evidence of a negative association between the viral load and WHZ.

**Table 1.** Predictors of Peak Viral Shedding Density

Covariate	*n*	Univariate Association With Peak Viral Load (95% CI)	*P* Value	Multivariate Association With Peak Viral Load (95% CI)	*P* Value
Sex, male	195	-.58 (-1.35 to .20)	.143	…	…
Age in months	195	-.04 (-.10 to .02)	.244	…	…
HIV exposed	195	.12 (-1.04 to 1.28)	.837	…	…
HIV infected	58	.58 (-3.55 to 4.71)	.779	…	…
Premature	195	.33 (-1.76 to 2.42 )	.755	…	…
Birth weight, kgs	195	.29 (-.34 to .93)	.365	…	…
Ever breast fed	195	-1.18 (-6.61 to 4.26)	.670	…	…
SAM	193	.16 (-1.05 to 1.37)	.793	…	…
WHZ	194	-.39 (-.63 to -.15)	.001	-.24 (-.49 to .01)	.060
WAZ	194	.09 (-.24 to .43)	.565	…	…
HAZ	190	.28(.12–.43)	.001	…	…
MUAC, cm	194	-.26 (-.56 to .04)	.089	…	…
Diarrhea episodes/day	195	…	…	…	…
1-3	…	REF	…	…	…
4-5	…	.61 (-.66 to .88)	.343	…	…
≥6	…	.70 (-.57 to 1.97)	.277	…	…
Diarrhea duration, days	195	…	…	…	…
1-4	…	REF	…	…	…
5	…	1.59 (-.01 to 3.20)	.052	…	…
≥6	…	-.45 (-2.12 to 1.22)	.596	…	…
Vomiting	195	…	…	…	…
Yes	…	1.81 (.33–3.29)	.017	…	…
Vomiting frequency/day	181	…	…	…	…
<5	…	REF	…	…	…
≥5	…	.07 (-.75 to .89)	.868	…	…
Vomiting duration, days	181	…	…	…	…
1	…	REF	…	…	…
2	…	.65 (-.61 to 1.91)	.310	…	…
≥6	…	1.33 (.15–2.52)	.028	…	…
Dehydration	195	…	…	…	…
None	…	REF	…	…	…
Some	…	.66 (-.52 to 1.84)	.271	…	…
Severe	…	1.31 (-.03 to 2.65)	.055	…	…
IV fluids	195	…	…	…	…
Yes	…	.57 (-.28 to 1.40)	.195	…	…
Oral fluids	195	…	…	…	…
Yes	…	.25 (-1.43 to 1.94)	. 767	…	…
Admission	195	…	…	…	…
Yes	…	.96 (.19–1.73)	.015	…	…
Outcome	195	…	…	…	…
Home	…	REF	…	…	…
Died	…	-2.05 (-5.89 to 1.80)	.294	…	…
Vesikari score	192	…	…	…	…
Mild	3	REF	…	REF	…
Moderate	23	8.27 (5.18–11.35)	.000	7.92 (4.96–10.90)	.000
Severe	167	8.46 (5.53–11.38)	.000	8.04 (5.23–10.84)	.000
IgA titre at presentation	163	-.27 (-.48 to -.07)	.010	-.21 (-.40 to -.02)	.032
Time from symptom onset	193	-.09(-.33 to .15)	.475	…	…

Abbreviations: HAZ, height for age Z score; HIV, human immunodeficiency virus; Ig, immunoglobin; IV, intravenous; MUAC, mid–upper arm circumference; REF, indicates reference category;SAM, severe acute malnutrition; WAZ, weight for age Z score; WHZ, weight for height Z score.

### Change in Viral Load Over Time and Duration of Viral Shedding: Intensive Cohort

**Figure 1.**
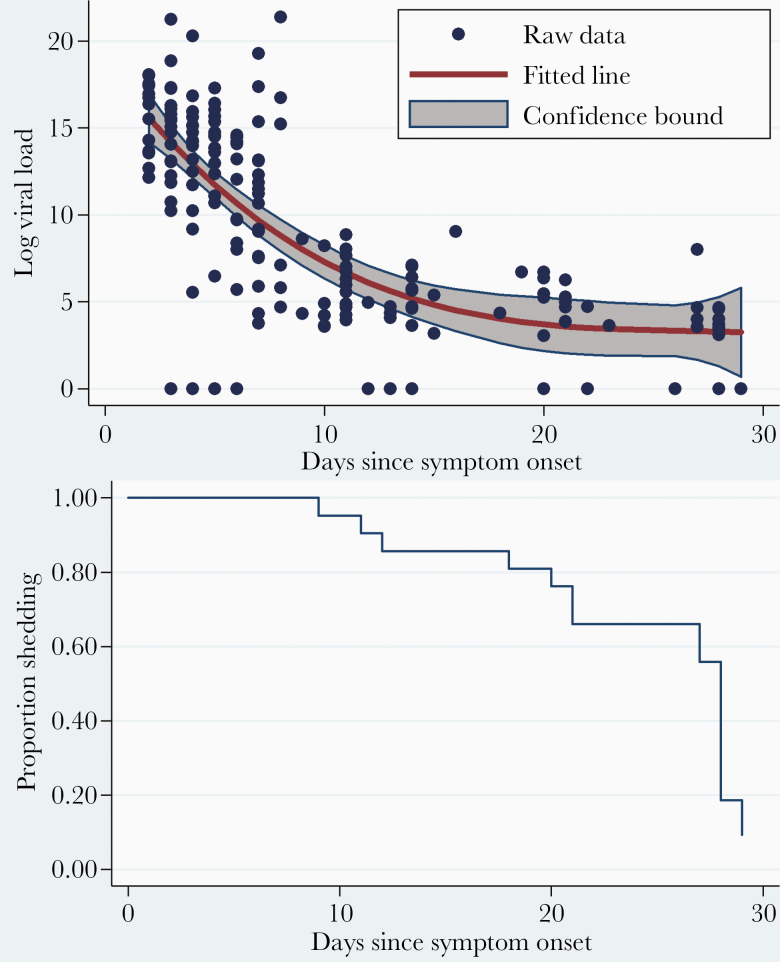
*A*, Rotavirus shedding over time in symptomatic children. The dots represent the raw data on log (viral load), and the red fitted line represents the regression line including a quadratic term, to account for the non-linear nature of viral decay. Confidence bounds represent the 95% confidence limits on either side of the fitted value. Regression coefficients are given in Supplementary Table S3. *B*, Kaplan Meier plot of time to cessation of shedding in index children. The analysis time is in days since symptom onset.

The viral loads ranged from 21 to 1.91 × 10^9^ copies. They declined significantly with the time from symptom onset (the regression coefficient for the relationship between log copy numbers and time in days since symptom onset, -1.68; 95% CI, -2.51 to -.85; Figure 1A; Supplementary Table S3). The viral loads were significantly higher when children were symptomatic (regression coefficient, 6.94; 95% CI, 5.15–8.73). This effect was reduced when adjusted for the time from symptom onset (regression coefficient, 1.87; 95% CI, .25–3.49). The proportion of children shedding rotavirus declined over time, from 100% at the first visit to 20% at the end of follow-up (Figure 1B). The median duration of shedding, based on a survival analysis, was 28 days (95% CI, 21–28; Figure 1B; Supplementary Table S4).

## DISCUSSION

In a cohort of vaccinated Malawian children with rotavirus gastroenteritis, children shed rotavirus in high densities at the time of initial symptom onset and continued to shed rotavirus for an extended period of time after symptom resolution. The fecal viral shedding density was positively associated with disease severity and negatively associated with anti-rotavirus IgA titres.

The pattern of rotavirus shedding observed among symptomatic rotavirus cases is similar to that reported among unvaccinated children from India [7]. In both Malawi and India, high viral titres on presentation rapidly declined over the first 10 days after symptom onset and then plateaued, with a median duration of shedding of approximately 4 weeks (28 days in Malawi, 24 days in India). This is substantially longer than the median duration of shedding reported from Australia (10 days) [6]. While this could reflect differences in the sensitivities of the assays used, the immune responses to both natural rotavirus infection and rotavirus vaccine are reduced in LICs, compared to higher-income settings [11]. The extended duration of shedding could, therefore, result from a delayed clearance of the replicating virus as a result of sub-optimal mucosal immunity, or very high rates of reinfection. A high frequency of asymptomatic rotavirus shedding has been described in young children from Malawi [12], and this may be partly explained by the prolonged fecal shedding of rotavirus following a symptomatic infection. Due to logistical restraints, the duration of follow-up in this study was limited, and the reported duration of shedding represents a minimum estimate. This is especially relevant considering our definition of cessation of shedding; it is possible that some of the children near the end of follow-up were misclassified as having stopped shedding.

Children who develop severe rotavirus disease despite being vaccinated shed large quantities of the virus. This may contribute to the ongoing community transmission of rotavirus and could partly explain the persisting high burden of disease in LICs, where indirect (herd) protection appears limited despite high vaccine coverage [4]. However, it is notable that recruitment into this study was biased towards those children with severe disease presenting to health-care facilities. In our cohort, disease severity was significantly associated with viral load; children with moderate and severe disease had significantly higher viral loads than those with mild disease. Immunity following rotavirus vaccination mimics that following natural rotavirus exposure, which generates incremental protection against rotavirus gastroenteritis of increasing severity [11]. It is therefore plausible that vaccination could reduce the severity of rotavirus gastroenteritis episodes and, thereby, decrease the total shedding burden of rotavirus in the community. The association between disease severity and viral load is most striking between mild and moderate/severe disease; however, the numbers of children recruited into this study with mild disease were small, presumably because most children with mild disease do not present to health-care facilities. The analysis is sensitive to the presence of children with mild disease, but robust to different analytical approaches. Further studies are required to ensure that children with mild disease are appropriately represented and to formally evaluate the potential for vaccination to reduce the viral shedding density at the population level.

Increasing anti-rotavirus IgA titres at presentation were negatively associated with fecal viral load, independent of disease severity. Serum anti-rotavirus IgA titres are known to correlate with intestinal IgA titres [13], and it is plausible that higher levels of intestinal IgA could reduce viral replication and, thereby, the fecal viral load. However, only a quarter of the vaccinated children in our cohort reached the criteria for seroconversion, and anti-rotavirus IgA titres are an imperfect correlate of protection, particularly in low-income settings [14].

We found that rotavirus viral shedding density is negatively associated with WHZ, implying that increasingly malnourished children shed more of the virus. Careful interpretation is required, as the standard deviations for our anthropometric measurements are outside the World Health Organization range for data quality assessment purposes [8]. Nevertheless, the negative association between WHZ and viral load is corroborated by weak evidence of a negative association between mid–upper arm circumference and viral load (Table 1). It is biologically plausible that children with poorer nutritional states could shed more rotavirus due to a reduced ability to mount mucosal immunity, differences in intestinal microbiome and mucosal integrity, and a tendency for more severe rotavirus disease in the presence of malnutrition [15].

### Conclusions

Children in Malawi shed large quantities of rotavirus for an extended period following an episode of moderate-to-severe diarrhea, despite prior vaccination. Persistently high fecal virus shedding may contribute to the high prevalence of asymptomatic infections in young children in the community and to ongoing rotavirus transmission. While reduced disease severity in Malawian children was associated with lower viral shedding density, the potential impact of this on population-level rotavirus transmission remains to be determined.

## Supplementary Data

Supplementary materials are available at The *Journal of Infectious Diseases* online. Consisting of data provided by the authors to benefit the reader, the posted materials are not copyedited and are the sole responsibility of the authors, so questions or comments should be addressed to the corresponding author.

jiz612_suppl_TableS1

jiz612_suppl_TableS2

jiz612_suppl_TableS3

jiz612_suppl_TableS4

## Notes

***Acknowledgments.*** The authors thank all infants and their families who participated, all members of the Rotavirus Response to Immunisation and Transmission Epidemiology (RotaRITE) study team, the Malawi Ministry of Health, and clinical staff at the recruitment sites. Aspects of this work have been presented at the European Society for Paediatric Infectious Diseases (ESPID) meeting, June 2018, Sweden.

***Disclaimer.*** The authors received no financial support or other form of compensation related to the development of the manuscript. The views expressed are those of the author(s) and not necessarily those of the National Health Service (NHS), the National Institute for Health Research, the Department of Health, or Public Health England.

***Financial support.*** This work was supported by Wellcome Trust Clinical PhD Fellowships (grant numbers 102466/Z/13/A to A. B. and 102464/Z/13/A to L. P.); the Wellcome Trust Programme (grant number 091909/Z/10/Z); the Malawi Liverpool Wellcome Trust (MLW) Programme Core Grant Strategic Award (grant number 101113/Z/13/Z); and the US National Institutes of Health (grant number R01-AI112970 to V. E. P.).

***Potential conflicts of interest.*** K. C. J. is supported by an International Wellcome Trust Training Fellowship (grant number: 201945/Z/16/Z). N. B.-Z. and K. C. J. have received research grant support from GlaxoSmithKline Biologicals for work on rotavirus vaccines. B. L. reports personal fees from Takeda Pharmaceuticals, personal fees from the Centers for Disease Control and Prevention Foundation, and personal fees from Hall Booth Smith PC, all unrelated to rotavirus vaccines. M. I.-G. has received research grant support from GlaxoSmithKline Biologicals and Sanofi Pasteur MSD for work on rotavirus; is affiliated with the National Institute for Health Research Health Protection Research Unit in Gastrointestinal Infections at University of Liverpool, in partnership with Public Health England and in collaboration with the University of East Anglia, University of Oxford, and the Quadram Institute; and is based at The University of Liverpool. N. A. C. has received research grant support and honoraria for participation in rotavirus vaccine advisory board meetings from GlaxoSmithKline Biologicals. V. E. P. is a member of the World Health Organization Immunization and Vaccine-Related Implementation Research Advisory Committee and has received reimbursement from Merck for travel expenses to attend a Scientific Input Engagement unrelated to rotavirus vaccines. All other authors report no potential conflicts.
