# Supplementary material for: Duration and Density of Fecal Rotavirus Shedding in Vaccinated Malawian Children With Rotavirus Gastroenteritis
Source: J Infect Dis. 2019 Dec 13;222(12):2035–40. doi: 10.1093/infdis/jiz612 (PMC7661767; doi:10.1093/infdis/jiz612)
Supplement: jiz612_suppl_TableS1 [file jiz612_suppl_tables1.docx]

Table S1. Characteristics of recruited children

|  | Summary statistic |
| --- | --- |
| Age in months (median and IQR) | 11.5(8.8,15.2) |
| Sex (male) (%) | 108/196 (55.1) |
| Diarrhoea (%) | 196/196 (100) |
| Duration (days) |  |
| 1-3 (%) | 173/196 (88.3) |
| 5 | 12/196 (6.1) |
| ≥6 | 11/196 (5.6) |
| Episodes(n)* |  |
| 1-4 (%) | 24/196 (12.2) |
| 5 | 86/196 (43.9) |
| ≥6 | 86/196 (43.9) |
| Vomiting (%) | 182/196 (92.9) |
| Duration (days) |  |
| 1 (%) | 23 (12.6) |
| 2 | 59 (32.4) |
| ≥3 | 100 (55.0) |
| Frequency (n) |  |
| <5 (%) | 123 (67.6) |
| ≥5 | 59 (32.4) |
| HIV |  |
| Exposed (%) | 25/196 (12.8) |
| Infected (%) | 3/89 (3.4) |
| Completed rotavirus vaccination (%) |  |
| Vaccinated (2 doses) | 194/196 (99.0) |
| Unvaccinated (0 dose) | 2/196 (1.0) |
| Time from vaccination to presentation in months (median and IQR) | 8.5 (5.9, 12.4) |
| Admitted (%) |  |
| Yes | 111/196 (56.6) |
| Vesikari score (IQR) | 14 (12, 16) |
| IgA titres at presentation, median (range) | 4 (0,831) |
| Dehydration (%) |  |
| None | 26/196 (13.3) |
| Some (5%) | 124/196 (63.3) |
| Severe (10%) | 46/196 (23.5) |
| IV fluids (%) |  |
| Yes | 58/196 (29.6) |
| Oral fluids (%) |  |
| Yes | 185/196 (94.4) |
| Outcome (%) |  |
| Home | 194/196 (99.0) |
| Died | 2/196 (1.0) |
| Anthropometry, mean (SD) |  |
| Adjusted WHZ | -0.59(1.61) |
| Adjusted WAZ | -0.46 (1.6) |
| Adjusted HAZ | -0.04 (2.46) |
| MUAC | 13.48 (1.28) |
| SAM | 23/194 (11.9) |
| Previous diarrhoeal presentation (%) | 91/196 (46.4) |
| Premature (%) | 7/196 (3.6) |
| Birth weight, mean (SD) | 2.96 (0.63) |
| Ever Breastfed (%) | 195/196 (99.5) |

*maximum number of episodes per day
