# Supplementary material for: Duration and Density of Fecal Rotavirus Shedding in Vaccinated Malawian Children With Rotavirus Gastroenteritis
Source: J Infect Dis. 2019 Dec 13;222(12):2035–40. doi: 10.1093/infdis/jiz612 (PMC7661767; doi:10.1093/infdis/jiz612)
Supplement: jiz612_suppl_TableS2 [file jiz612_suppl_tables2.docx]

**Table S2. Association between Vesikari score and peak viral load – sensitivity analysis**

| Covariate | N | Univariate association with peak viral load (95% CI) | P value |
| --- | --- | --- | --- |
| Vesikari* | 192 | 0.237 (0.1, 0.38) | 0.001 |
| Vesikari (categorical)** |  |  |  |
| ≤12 | 57 |  |  |
| 13-14 | 56 | 0.89 (-.11, 1.90) | 0.083 |
| 15-20 | 80 | 1.15 (0.22, 2.10) | 0.016 |

*continuous variable **divided into 3 approximately equal groups.
