# Supplementary material for: Duration and Density of Fecal Rotavirus Shedding in Vaccinated Malawian Children With Rotavirus Gastroenteritis
Source: J Infect Dis. 2019 Dec 13;222(12):2035–40. doi: 10.1093/infdis/jiz612 (PMC7661767; doi:10.1093/infdis/jiz612)
Supplement: jiz612_suppl_TableS3 [file jiz612_suppl_tables3.docx]

Table S3. Regression model for shedding curve

| Log viral load | Regression coefficient | P value | 95% Confidence limits |
| --- | --- | --- | --- |
| Time since symptom onset (TS)* | -1.68 | <0.001 | -2.51, -0.85 |
| TS^2^* | 0.06 | 0.071 | -0.01, 0.13 |
| TS^3^* | -0.01 | 0.319 | -0.00, 0.00 |

*Where TS=time in days from symptom onset, TS^2^= quadratic term of TS, TS^3^ is cubic term of TS
