# Supplementary material for: Duration and Density of Fecal Rotavirus Shedding in Vaccinated Malawian Children With Rotavirus Gastroenteritis
Source: J Infect Dis. 2019 Dec 13;222(12):2035–40. doi: 10.1093/infdis/jiz612 (PMC7661767; doi:10.1093/infdis/jiz612)
Supplement: jiz612_suppl_TableS4 [file jiz612_suppl_tables4.docx]

Table S4. Survivor functions for Kaplan Meir analyses

| Time | Total at beginning of time period | Events | Complete follow up (event free) | Survivor function | Standard error | 95% confidence interval |
| --- | --- | --- | --- | --- | --- | --- |
| 9 | 21 | 1 | 0 | 0.95 | 0.047 | 0.707, 0.993 |
| 11 | 20 | 1 | 0 | 0.90 | 0.064 | 0.670, 0.975 |
| 12 | 19 | 1 | 0 | 0.86 | 0.076 | 0.620, 0.952 |
| 18 | 18 | 1 | 0 | 0.81 | 0.086 | 0.569, 0.924 |
| 20 | 17 | 1 | 1 | 0.76 | 0.093 | 0.519, 0.893 |
| 21 | 15 | 2 | 0 | 0.66 | 0.105 | 0.416, 0.822 |
| 27 | 13 | 2 | 2 | 0.56 | 0.111 | 0.322, 0.742 |
| 28 | 9 | 6 | 1 | 0.19 | 0.095 | 0.047, 0.396 |
| 29 | 2 | 1 | 1 | 0.09 | 0.081 | 0.008, 0.315 |
